# Altered third‐party punishment in Huntington's disease: A study using neuroeconomic games

**DOI:** 10.1002/brb3.1908

**Published:** 2020-10-18

**Authors:** Martin Brüne, Sarah Maria von Hein, Christian Claassen, Rainer Hoffmann, Carsten Saft

**Affiliations:** ^1^ Department of Psychiatry, Psychotherapy and Preventive Medicine Division of Social Neuroscience and Evolutionary Medicine LWL University Hospital Bochum Ruhr University Bochum Bochum Germany; ^2^ Department of Neurology Huntington Centre NRW St. Josef‐Hospital Ruhr University Bochum Bochum Germany; ^3^ Klinikum Osnabrück Klinik für Neurologie und neurologische Frührehabilitation Osnabrück Germany

**Keywords:** “theory of mind”, economic decision‐making, Huntington's disease, rules of social exchange, social reward

## Abstract

**Background:**

Huntington's disease (HD) is a heritable degenerative brain disease caused by a mutation in the huntingtin gene with excessive repeats of the base triplet cytosine–adenine–guanine (CAG), which codes for the aminoacid glutamine. HD is associated with a broad spectrum of neurocognitive dysfunction, including deficits in social cognition. The appreciation of fairness rules and reciprocity has not been studied in HD. Based on theoretical considerations suggesting that brain regions known to be affected from HD are involved in economic decision‐making, the present study sought to examine HD patients' performance in two neuroeconomic games.

**Methods:**

Twenty‐nine manifest HD mutation carriers (20 males, nine females) performed an Ultimatum Game (UG) and a Dictator Game (DG) where third‐party punishment of observed unfairness was required. In addition, patients were tested for neurocognition and the ability to understand other people's mental states (“theory of mind”). For comparison, a clinical control group of 30 patients with chronic schizophrenia, and 30 unaffected healthy controls matched for age and verbal intelligence took part in the study.

**Results:**

Patients with HD had some appreciation of fairness rules, as they tended to reject unfair offers in the UG similar to controls. However, unlike the other two groups, individuals with HD did not punish observed unfairness from a third‐party perspective. This lack of “altruistic punishment” was associated with deficits in executive functioning including working memory, inhibitory control and cognitive flexibility, and to a lesser degree with poor “theory of mind.”

**Conclusions:**

HD seems to be associated with impairments in understanding of more complex rules of social exchange. Aside from deficits in executive functioning, this behavior could, in part, be linked to an inability to experience third‐party punishment as rewarding.

## Graphical abstract

Huntington's disease (HD) is a heritable degenerative brain disease associated with a broad spectrum of neurocognitive dysfunction. Here, we studied patients' understanding of rules of social exchange using neuroeconomic games that required an understanding of unfairness. Interestingly, individuals with HD did not punish observed unfairness from a third‐party perspective, which was associated with deficits in executive functioning and to a lesser degree with poor “theory of mind.”

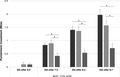

## 1 INTRODUCTION

Huntington's disease (HD) is a severe degenerative brain disease that is characterized by abnormal involuntary movements and a broad range of cognitive and behavioral signs and symptoms, some of which can predate the onset of chorea. HD is monogenetically inherited with a penetrance of 100 percent, affecting 5–7 in 100,000 people. The course and onset of HD seems, in part, associated with the number repeats of the base triplet cytosine–adenine–guanine (CAG), which codes for the aminoacid glutamine, whereby high repeat numbers are generally linked to an earlier onset and faster disease progression (Langbehn et al., 2004; Walker, 2007).

With regard to non‐motor symptoms, cognitive deterioration in the course of HD is an obligatory sign, but there is also abundant evidence for difficulties in executive action control, working memory, verbal fluency, altered reward processing, and social cognitive deficits (Beste et al., 2010; Bodden et al., 2010; Bora et al., 2016; Snowden et al., 2003; Watkins et al., 2000).

“Social cognition” is an umbrella term for several independent domains including social perception, facial emotion recognition, mentalizing or “theory of mind,” and attributional style (e.g., Kennedy & Adolphs, 2012). In one of the first studies, Sprengelmeyer et al. (1996) discovered that patients with HD are specifically impaired in recognizing the facial expression of disgust, while other studies have corroborated the finding that deficits in emotion processing occur at all stages of the disease (Henley et al., 2008; Johnson et al., 2007; Novak et al., 2012; Snowden et al., 2008). Similarly, research has consistently shown that patients with HD have difficulties in appreciating other people's mental states across different modalities such as humor perception, false belief understanding, recognition of cooperation and deception, or “faux‐pas” comprehension (Brüne et al., 2011; Eddy et al., 2011; Havet‐Thomassin et al., 2008; Snowden et al., 2003). These social cognitive impairments in HD are not entirely independent of neurocognitive functioning, particularly inhibitory control, but also forward planning, judgement, and reasoning (reviewed in Gleichgerrcht et al., 2010). In fact, Brüne et al. (2011) found that the association of “theory of mind” deficits with executive functions in patients with HD strikingly resembled the pattern found in chronic schizophrenia, a group of disorders also sharing some clinical features with HD. Together, social cognitive impairment and executive dysfunction are thought to substantially contribute to behavioral symptoms and interpersonal problems in HD (Bora et al., 2016).

Another area of research that has received increasing attention with regard to HD is the ability to process reward and punishment‐related stimuli. In fact, there is good reason to propose that individuals with HD have profound difficulties in this respect. Palminteri et al. (2012) were among the first to demonstrate, in a functional brain imaging study, that pre‐manifest mutation carriers and manifest subjects with HD are specifically impaired in avoiding punishment in an experimental condition that aims at maximizing monetary gains, while minimizing the risk of losing (virtual) money. Put differently, reward learning was intact in HD subjects, while they exhibited a selective deficit in punishment learning. Somewhat contradictory to this finding, Enzi et al. (2012) reported that reward processing, but not punishment processing, was altered in pre‐manifest carriers of the HD mutation. Specifically, individuals who were categorized as being close to the onset of motor symptoms activated the ventral striatal area less in the reward condition compared to a control condition, whereas no such difference occurred between punishment and control condition. Moreover, this activation pattern was absent in individuals who were still further away from the anticipated onset of motor symptoms (Enzi et al., 2012). These results are interesting, because degeneration of the striatum is central to the neuropathology of HD, and because the ventral striatum is specifically involved in reward processing, while a dorsal stream linked to areas of the limbic system such as the anterior insula may be more relevant for punishment‐associated cue processing, and it may well be that the ventral and the dorsal stream are differentially affected over the course of the disease (Enzi et al., 2012; Palminteri et al., 2012).

### 1.1 Aims and hypotheses

Given that individuals with HD have difficulties in appreciating the mental states of others and in processing stimuli associated with reward or punishment, we sought to examine economic decision‐making tapping into two different domains: one is the perception of unfairness toward oneself, the other concerns one's motivation to engage in third‐party punishment. In fact, humans are generally highly sensitive toward social inequity or unfairness, and tend to invest own resources—in contrast to the idea of *homo oeconomicus*—in re‐installing equity even among unrelated others (de Quervain et al., 2004). The latter has been referred to as “third‐party punishment” or “altruistic punishment” (Fehr & Gächter, 2002) and it is known to be associated with the experience of reward (de Quervain et al., 2004). Accordingly, we predicted aberrant behavioral performance of HD patients in the economic games, and, as in previous work, impaired “theory of mind.” We further hypothesized that economic decision‐making in HD would correlate with patients' performance on a “theory of mind” task, and with executive functioning such as working memory, cognitive flexibility, and inhibitory control. Based on the afore‐mentioned previous similarities in social cognitive task performance between patients with HD and individuals with chronic schizophrenia, we decided to include a group of patients with schizophrenia, which gave us the opportunity to examine whether putative alterations in task performance in the economic games were specific to one or the other clinical group.

## 2 METHODS

### 2.1 Participants

Twenty‐nine patients (20 males, nine females) with genetically confirmed and manifest HD (diagnostic confidence level 4) were included in the present study. Patients' mean age was 49.5 years (range 32–69 years; *SD* 8.9), with an average duration of motor symptoms of 3.3 years (range 0–14 years; *SD* 3.4). Patients' verbal IQ was 102 (*SD* 16.1). The total functional capacity as determined using the Unified Huntington's Disease Rating Scale (UHDRS) was 10.4 (*SD* 1.9) with an independence score (IS) of 80.4 (*SD* 9.5) and Motor Score (MS) of 32.2 (*SD* 13.4). Most patients received psychotropic medication. That is, 19 were on tiapride or tetrabenazine, 12 patients took selective serotonin reuptake inhibitors, five others took mirtazapine or duloxetine, 11 received second‐generation antipsychotics, and one patient was on low‐dose benperidol.

For comparison, we recruited a clinical control group of 30 patients (20 males, 10 females) with chronic schizophrenia, diagnosed according to DSM‐IV criteria. Specifically, the schizophrenia group met the criteria of “deficit schizophrenia” (Bryson et al., 2001; Carpenter et al., 1988), characterized by pervasive negative symptoms over at least 12 months, and a duration of psychosis of more than 8 years (average duration of illness 19.3 years; range 3–42 years; *SD* 9.2). The schizophrenia group had an average age of 42.8 years (range 21–62 years; *SD* 10.3) with a verbal IQ of 101 (*SD* 13.4). Disease severity according to the Positive and Negative Syndrome Scale (PANSS) was 67.8 (*SD* 22.0) points, indicating moderate illness activity. All schizophrenia patients received a stable dose of second‐generation antipsychotics.

In addition, 30 healthy subjects (10 males, 20 females) were recruited from the general public and the local university. The mean age of the healthy control group was 42.8 (range 22–69 years; *SD* 13.8) with a mean verbal IQ of 108 (*SD* 15.4). Exclusion criteria across groups comprised a history of drug abuse (except for tobacco), severe neuropsychiatric conditions (other than HD or schizophrenia), mental retardation, and insufficient knowledge of German language. All participants gave informed consent in writing. The study was approved by the Ethics Committee of the Medical Faculty of Ruhr University Bochum, Germany.

Demographic characteristics of the participants and ratings of psychopathology, as well as number of CAG repeats in the HD group, are shown in Table 1.

**TABLE 1:** Demographic data and psychopathology ratings of patients with HD, schizophrenia, and controls

	HD	schizophrenia	controls
*N*	29	30	30
M:F	20:9	20:10	10:20
Age	49.5 (8.9)	42.8 (10.3)	42.8 (13.8)
Duration of illness	3.3 (3.4)	19.3 (9.2)	‐‐‐
Verbal IQ	102 (16.1)	101 (13.4)	108 (15.4)
MSAT sequencing	21.9 (7.4)*§	26.6 (8.2)*	32.3 (4.8)
MSAT questionnaire	19.7 (3.4)*	18.2 (5.2)*	22.3 (1.3)
MSAT total score	41.6 (9.7)*	44.8 (12.1)*	54.6 (5.6)
UHDRS TFC	10.4 (1.9)	—	—
UHDRS IS	80.4 (9.5)	—	—
UHDRS CS	207.1 (65.4)	—	—
UHDRS MS	32.2 (13.4)	—	—
CAG repeats	43.9 (2.6)		
PANSS positive		18.7 (7.2)	
PANSS negative		17.1 (8.0)	
PANSS global	—	32.0 (13.0)	—
PANSS sum score	—	67.8 (22.0)	—

Note

Significant differences between either one of the clinical groups and controls are indicated by an “*,” differences between the clinical groups are marked by a “§.”

Abbreviations: CAG, cytosine, adenine, guanine; CS, Cognitive Score; F, female; IS, Independence Score; M, male; MS, Motor Score; MSAT, Mental State Attribution Task (“theory of mind”); PANSS, Positive and Negative Syndrome Scale; TFC, Total Functional Capacity; UHDRS, Unified Huntington Disease Rating Scale.

### 2.2 Economic games

The economic games used in the present study were adapted versions of the ones used by Wischniewski and Brüne (2011) and were shortened to 12 trials in each game to not overburden the patients' attention span. The original version comprised 44 trials (i.e., 11 per condition), because it was utilized in EEG research. Prior to testing, all participants received written instructions and performed a practice trial. They were also informed that the images of the other players in the Ultimatum Game (UG) and the Dictator Game (DG) were placeholders of individuals who in previous rounds showed exactly this kind of behavior. Participants received 10 Euros and another 0–5 Euros depending on their actual performance in the economic games. They were informed about the possibility of gaining additional money, but were left oblivious to the exact mathematical procedure, and thus did not know whether altruistic or selfish behavior was rewarded. In fact, participants received an additional 10 percent of the money invested in third‐party punishment in the DG; hence, altruistic attitudes were rewarded.

#### 2.2.1 Ultimatum game

In the UG, two players were asked to split an amount of 10 money units (MU), whereby a virtual character served as proposer how to share the money, while the participant acted as the recipient. There were 12 trials altogether, three of which involved fair splits (i.e., 5:5), the other nine trials were randomly presented and reflected different degrees of unfairness (i.e., 7:3, 8:2, and 9:1, respectively). The participant in the role of the recipient had two options: to reject the offer (in that case, neither of the players received any MU; thus, rejection of an offer reflects a mild form of punishment), or to accept the offer as proposed. The response was given by mouse click.

#### 2.2.2 Dictator game with punishment option

In the DG, participants acted as a third person observing two other players (a proposer and a recipient, just like in the UG) sharing 10 MU. In contrast to the UG, the recipient had no choice as to accept every offer made by the proposer. The participant had the option to invest own MU to punish the proposer for his or her unfair behavior. For every 0.5 MU invested, the proposer's amount was reduced by 1 MU, while the recipient's amount increased by 1 MU. For example, if the virtual proposer suggested to keep 8 MU for himself and give 2 MU to the recipient, the participant could invest 1.5 MU to induce equity (in this example, the MU of the proposer would have been reduced by 3 MU, while the sum of the recipient would have increased by 3 MU).

Like in the UG, there were a total of 12 trials with three trials per split condition (5:5, 7:3, 8:2, and 9:1). The trials were again presented in random order. First, the participant viewed facial images of the two players (proposer and recipient). Subsequently, the proposer's actual offer to the recipient was depicted on the screen. Next, the participant was asked whether or not he or she would like to change the distribution by investing own MU. The distribution of money units was visualized using a slide bar and stacks of money units. Sliding the mouse cursor to the left or right changed the distribution in real time, such that no mathematical calculation was necessary. Finally, the participant confirmed the invested amount with a mouse click.

### 2.3 Theory of mind

The ability to appreciate others' mental states was examined using a theory of mind cartoon task or “Mental State Attribution Task” (MSAT). The MSAT has widely been used by our and other study groups, including research in HD (e.g., Abdel‐Hamid et al., 2009; Brüne et al., 2011). It comprises six cartoon stories, two of which show a scenario where two characters cooperate with one another, two cartoons depict a scenario where one character deceived a second character, and another two cartoons illustrate a scenario where two characters cooperate to deceive a third. Each cartoon story consists of four cards (an exemplary cartoon is shown in Figure S1).

The cards were presented in jumbled order on a computer screen. First, participants were asked to order them in a logic sequence of events by clicking on the symbols on the screen which changed the sequence of cards. Participants could make as many moves as they wanted until they approved their decision by mouse click. Two points were given for the first and last correctly sequenced cards, and one point each for correct sequencing the two middle cards (thus 6 pts. maximum per picture story, max. sum score 36 pts.). Two practice cartoons were taken from Langdon et al.'s picture stories and presented prior to the MSAT (Langdon et al., 1997).

In addition to the sequencing task, participants were asked to respond to twenty‐three questions, which directly asked for the participants' comprehension of the cartoon characters' mental states (for example, “What do you think the person (pointing to the respective character) intends to do?”). Overall, questions addressed the participants' ability to recognize cooperation, deception, to detect cheating and to comprehend true and false beliefs of the characters in the picture stories. A total score of sequencing and questionnaire was calculated (59 pts. maximum).

### 2.4 Neuropsychological tasks

General intelligence was estimated using the “Mehrfachwahlwortschatztest” (MWT‐B), which may best be translated as “Multiple Choice Verbal Comprehension Test.” In clinical populations, the MWT‐B is believed to reflect premorbid intelligence (Lehrl, 2005).

In the HD group, disease severity was examined using the Unified Huntington Disease Rating Scale (UHDRS), which, aside from a motor score, included the symbol digit test, verbal fluency, color naming, color reading, and stroop interference; these were summarized as a Cognitive Score (CS) reflecting, in part, executive functioning. Total Functional Capacity (TFC) and Independence Scale (IS) as part of the UHDRS were also assessed (Huntington Study Group, 1996). In the schizophrenia group, disease severity was measured using the Positive and Negative Syndrome Scale (PANSS; Kay et al., 1987).

### 2.5 Statistical analysis

Statistical analysis was carried out using the Statistical Package for the Social Sciences (SPSS), Version 26 for Windows. Our analyses were based on previous publications dealing with mean count data (Ridout et al., 1998). Accordingly, we determined the percentage of the mean acceptance rate per condition in the UG (i.e., we used the mean of the acceptance rates of the four trials per condition) and the mean invested MU for each split condition in the DG. To compare differences in performance, we calculated separate general linear models (GLM) (i.e., multivariate analyses of variance, MANOVAs) for the two games with the four conditions (5:5, 7:3, 8:2, and 9:1) as the dependent variables (DVs), and diagnoses as independent variables (IVs). We also report results of ANOVAs comparing groups for each separate DV, if the GLM showed a significant effect. This way of analysis was chosen, because the data deviated from normality in some conditions, and MANOVAs are considered fairly robust against violations of normality. Finally, wherever performance in the UG or DG was significantly different between groups, we calculated additional MANCOVAs, and ANCOVAs, with “theory of mind” as a co‐variate. In addition, generalized linear models with repeated‐measures designs allow the observation of direct interaction effects. So, we also report the interaction of neuroeconomic performance (fitted into the equation as within‐subject factors) and diagnosis (fitted into the equation as between‐subject factor), because this approach has also been used for the analysis of neuroeconomic approaches. Because most clinical measures used ordinal scales, non‐parametric correlation analyses were used, whereby alpha was adjusted to 0.01, given the number of correlations.

## 3 RESULTS

### 3.1 Between‐group differences

The groups differed with regard to age (*F* = 3.45, *df* = 2, *p* = .036), with the group of HD patients being older than the other two groups. Post hoc comparisons (Bonferroni‐corrected) failed, however, to reach statistical significance (*p* = .075). There was no statistically significant difference between the groups regarding verbal IQ (*F* = 2.23, *df* = 2, *p* = .114).

When looking at MSAT performance, between‐group ANOVAs showed that both patient groups performed more poorly on the sequencing task than the healthy control group (*F* = 16.9; *df* = 2; *p* < .001), the questionnaire part (*F* = 9.47; *df* = 2; *p* < .001), and hence obtained lower total scores than the control group on the MSAT (*F* = 15.2; *df* = 2; *p* < .001). Patients with HD made more errors in the sequencing part of the MSAT than the two other groups, and post hoc comparisons confirmed that the difference between HD patients and schizophrenia patients remained significant in this regard (*p* = .032). In contrast, no differences emerged regarding the questionnaire part and the total score of the MSAT between the clinical groups (*p* = .371, and *p* = .575, respectively).

Since symptom severity in the HD and the schizophrenia group was measured using different scales, a meaningful comparison was precluded. Similarly, cognitive performance was tested in greater detail only in the HD group.

**FIGURE 1:**
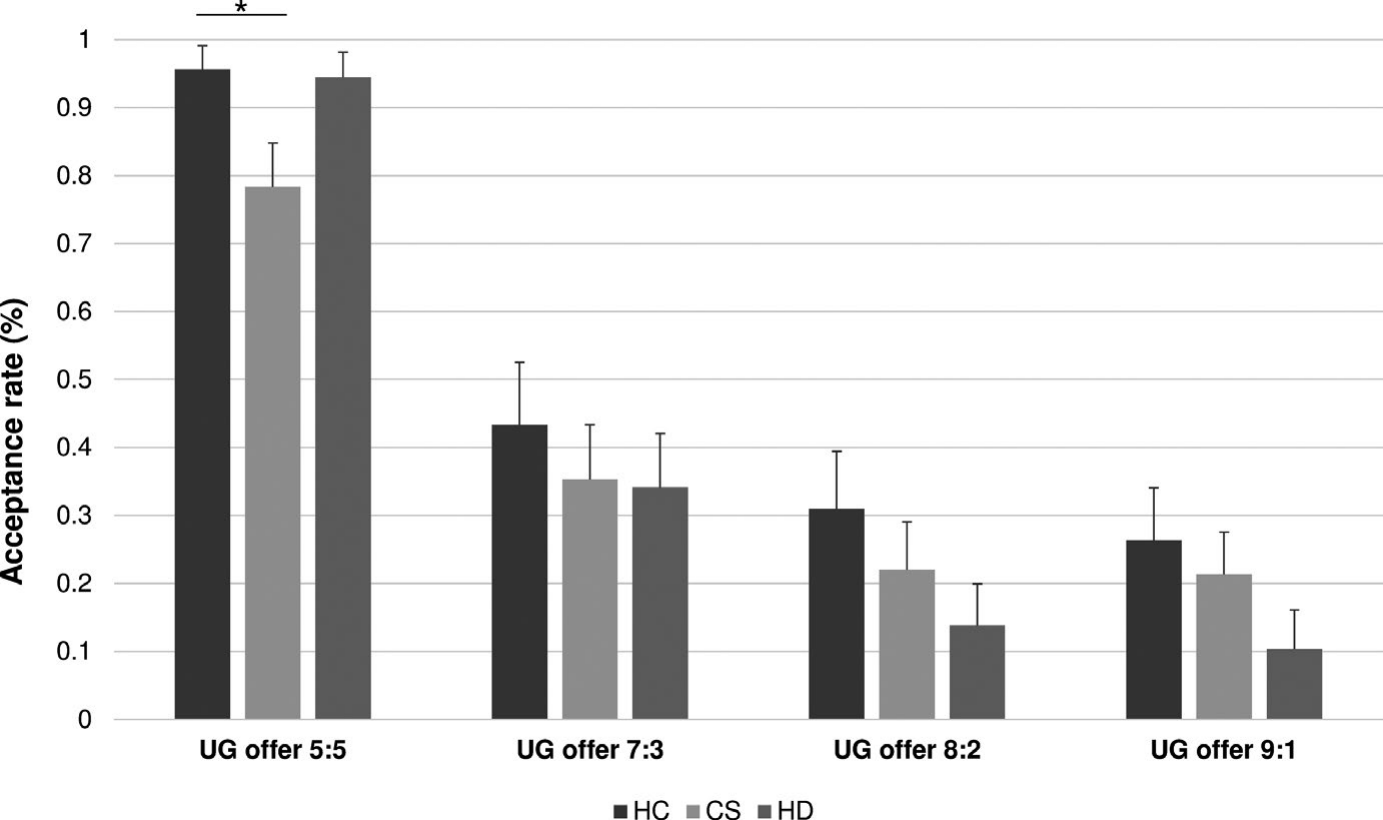
Performance of patients with HD, schizophrenia patients and controls in the Ultimatum Game (UG). Bars showing the acceptance rates for fair and unfair offers (in percent)

As regards acceptance rates in the UG, a MANOVA with the four split conditions as DVs and diagnosis as the independent variable (IV) showed no significant effect (*F* = 1.590; *df* = 8; *p* = .131). Moreover, the GLM repeated‐measures design revealed no interaction between performance in the UG and diagnosis (Greenhouse–Geisser correction applied due to violation of sphericity; *F* = 1.682, *df* = 4.565, *p* = .147). Overall, there was a decline in acceptance of offers with increasing unfairness in all groups (Figure 1). An exploratory between‐group ANOVA revealed, however, that patients with schizophrenia accepted fair offers significantly less often than patients with HD and healthy controls (*F* = 4.334; *df* = 2; *p* = .016). Post hoc Bonferroni correction confirmed a significant difference between schizophrenia patients and healthy controls for the acceptance rate of fair offers (*p* = .029). No group differences emerged for the unfair conditions. Moreover, as the clinical groups performed more poorly in the MSAT, an ANCOVA controlling for “theory of mind” performance remained significant for the fair split condition (*F* = 4.526; *df* = 4; *p* = .014).

**FIGURE 2:**
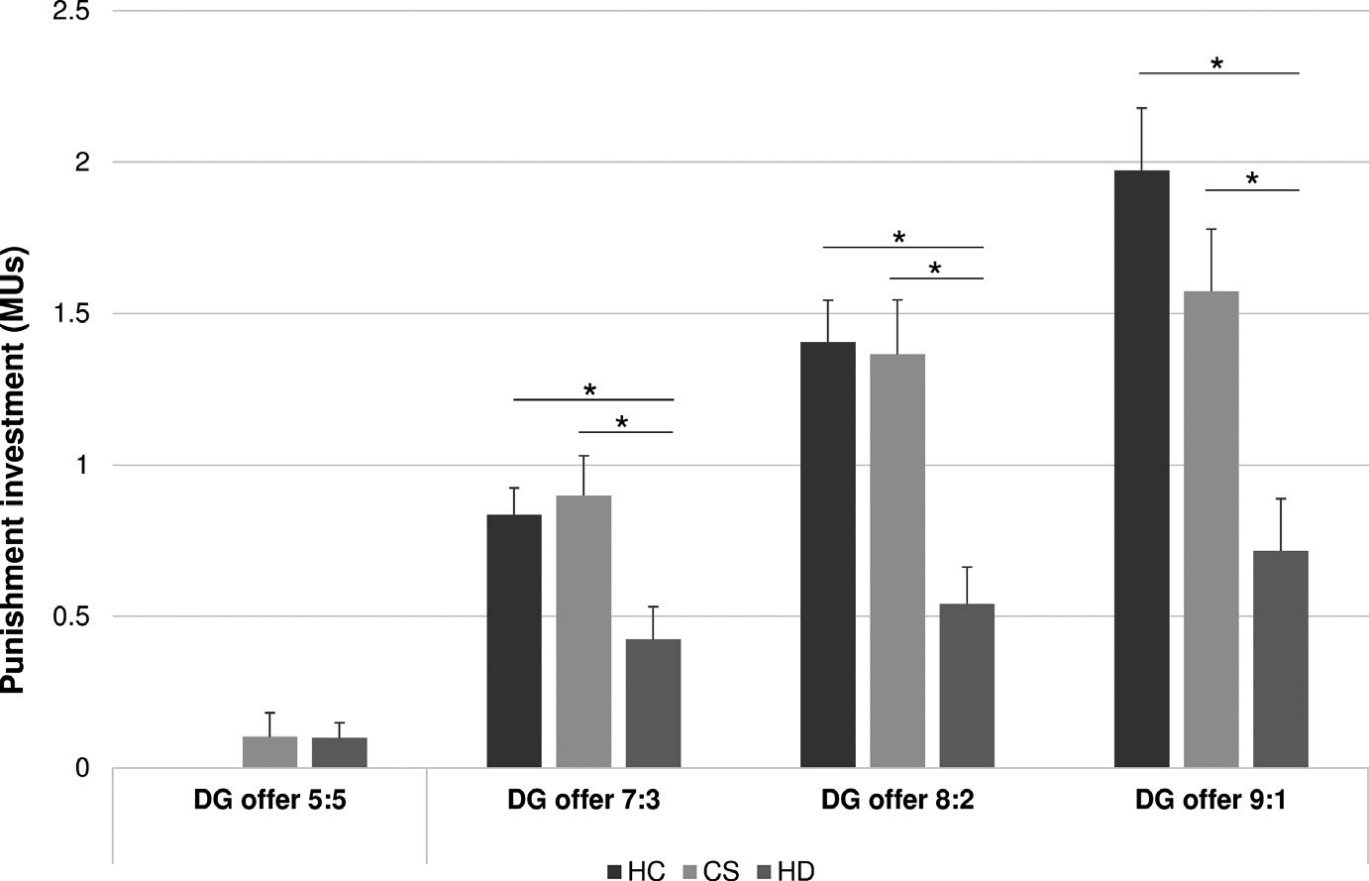
Performance of patients with HD, schizophrenia patients, and controls in the Dictator Game (DG). Bars illustrating the investment (in MUs) in third‐party punishment. Note that punishment of the fair condition was virtually absent in all control subjects, which is why the bar for this group is not visible

In contrast to the largely inconspicuous behavior in the UG, a MANOVA with punishment investments for each condition as the DVs and diagnosis as the IV revealed a significant overall effect of diagnosis (*F* = 4.694; *df* = 8; *p* < .001). Importantly, The GLM repeated‐measures analysis revealed a significant interaction between behavior in the DG and diagnoses (Greenhouse–Geisser corrected *F* = 11.198, *df* = 3.104, *p* < .001). Overall, there was an incremental punishment investment with increasing unfairness (Figure 2). While between‐group ANOVAs showed no difference for the fair condition (i.e., no punishment of fairness) (*F* = 1.234; *df* = 2; *p* = .296), significant group differences occurred for all unfair conditions (i.e., 7:3 condition: *F* = 5.458; *df* = 2; *p* = .006; 8:2 condition: *F* = 10.406; *df* = 2; *p* < .001; 9:1 condition: *F* = 10.459; *df* = 2; *p* < .001). Post hoc comparisons (Bonferroni‐corrected) revealed that in all unfair split conditions, HD patients invested significantly fewer MUs compared to the other two groups (all individual comparisons with *p* < .05), with the most significant effects for the two most unfair conditions (*p* < .01), whereas no difference emerged between schizophrenia patients and controls (all p‐values> 0.05). A MANCOVA controlling for “theory of mind” continued to show significant group differences in third‐party punishment (*F* = 3.615; *df* = 8; *p* = .001). Moreover, between‐group ANOVAs remained significant for the unfair conditions when controlling for “theory of mind” (all *p* < .003). We also looked at “age” as a potential between‐group difference (though the difference was only marginal; see above). However, neither did age change the findings with regard to UG performance, nor third‐party punishment in the DG (i.e., there was no statistical effect of age on altruistic punishment; all p‐values remained virtually the same).

### 3.2 Correlations within the HD group

To examine the association of task performance in the UG and the DG with neurocognitive functioning, we performed Spearman‐rho non‐parametric correlation analyses in the HD group (because of the ordinal scale characteristics of neurocognitive tasks). Note that the p‐value was adjusted to <0.01 to reduce the risk of Type‐I error.

Most interestingly, there were no correlations between the acceptance rates in the UG (with one single exception) with neurocognition, but several correlations between behavior in the DG and neurocognition, especially the symbol digit test (*r*_s_ = .541; *p* = .002), and the color reading with the most unfair condition (*r*_s_ = .497; *p* = .001), indicating that better executive functioning correlated with greater investment in the DG. In addition, a high correlation emerged between the total MSAT score and neurocognition, that is,. CS (*r*_s_ = .717; *p* < .001). Partial correlation analyses revealed that the correlations between neurocognition and DG performance remained significant when “theory of mind” was partialled out, but failed to reach the stricter significance level of 0.01 (partial correlation between punishment investment in the 9:1 condition with the Cognitive Score of the UHDRS: *r*_s_ = .409; *p* = .031), whereas the correlation between punishment investment in the 9:1 condition with “theory of mind,” controlled for neurocognition, was not significant (*r*_s_ = .038; *p* = .846). Together this suggests an association of third‐party punishment with neurocognition (particularly executive function).

## 4 DISCUSSION

The present study sought to address the question whether individuals with HD were impaired in their ability to appreciate rules of social exchange. Specifically, based on anatomical and behavioral considerations suggesting that the brain network involved in economic decision‐making is affected by the disease process (Enzi et al., 2012; Palminteri et al., 2012; de Quervain et al., 2004), we hypothesized that patients with HD would present aberrant performance in neuroeconomic games. In line with predictions, HD patients invested fewer MUs in third‐party punishment, while they performed similarly in another game requiring a response to another's unfairness. These findings are highly interesting for a number of reasons. First, in the UG patients with HD showed fairly typical behavior of rejecting offers with increasing unfairness (reviewed in Wischniewski and Brüne (2011)). That is, the most unfair offer was rejected most, while milder unfairness was slightly more tolerated. Interestingly, in the clinical control group of patients with schizophrenia, a bizarre finding was that fair offers were rejected by some patients, which did not occur in the HD or the healthy control group. In any event, the findings from the UG indicate that a basic understanding of fairness and unfairness seemed to be preserved in HD. Moreover, the recognition of unfairness was independent of “theory of mind” or age.

In contrast to the inconspicuous behavior of HD patients in the UG, their performance in the DG differed greatly from the other two groups. Indeed, individuals with HD engaged significantly less in third‐party punishment compared to patients with chronic schizophrenia (who performed similarly to controls) and with healthy controls. These differences remained statistically significant when controlling for “theory of mind,” suggesting that the aberrant behavior in the DG was independent of patients' difficulties in appreciating others' mental states. In fact, both clinical groups (HD and schizophrenia) performed more poorly than controls on a “theory of mind” task, but differed only mildly from one another in this regard. In other words, since patients with schizophrenia displayed difficulties in appreciating others' mental states, but engaged in third‐party punishment similar to controls, it is not plausible to assume that the poorer performance of HD patients in “theory of mind” accounted for their lack of third‐party punishment. Instead, in the HD group some correlations occurred between economic decision‐making in the DG (but not in the UG) and with executive functioning, indicating that poor inhibitory control may have contributed to the absence of third‐party punishment. This finding is entirely in line with Gleichgerrcht et al.'s (2010) review describing a strong link between decision‐making and executive functioning in HD, even though the review did not primarily concern economic decision‐making. Unfortunately, this putative association was not examined in the clinical control group.

Given that the anatomical substrate of HD involves subcortical (i.e., ventral and dorsal striatal (Petrasch‐Parwez et al., 2012) as well as limbic and cortical structures such as the DLPFC (Enzi et al., 2012; Gleichgerrcht et al., 2010; Palminteri et al., 2012; Wolf et al., 2008), it could be that disruption of these brain circuits causes alterations in economic decision‐making (de Quervain et al., 2004; Wischniewski et al., 2009). However, this interpretation is somewhat contradictory to experimental brain research utilizing repetitive transcranial magnetic stimulation (rTMS) or transcranial direct current stimulation (tDCS). By and large, studies using economic games have demonstrated that a functional disruption of the DLPFC by either rTMS or tDCS leads to greater acceptance of unfair offers in the UG, and to greater third‐party punishment (overview in Brüne et al. (2012)), which was not observed in the HD group. However, other research using brain stimulation techniques has suggested that another function of the DLPFC could be to execute control over selfish motives (Knoch et al., 2008; Müller‐Leinß et al., 2018), which would, in part, be more compatible with the findings of the present study concerning patients' behavior in the DG, but not the UG.

Another possible explanation for the present findings concerns alterations of reward and punishment processing (Enzi et al., 2012; Palminteri, et al., 2012). Findings from functional brain imaging studies suggest that both reward and punishment learning might be impaired in HD, and social reward seems to involved the same neural network as monetary reward (Wake & Izuma, 2017). While reward and punishment processing involves striatal brain areas, a speculative question is whether deficits in reward or punishment‐associated learning could translate into aberrant engagement in third‐party punishment. Indeed, prosocial behavior, including altruistic deeds like charitable donations, is associated with the experience of reward, which is accompanied by striatal activity in neuroimaging studies (Moll et al., 2006). This could, in turn, suggest that a lack of experiencing reward may cause individuals with HD to abstain from third‐party punishment (as shown in the DG), even though they may well comprehend unfairness per se (and act upon it, if unfairness is directed toward themselves). It would therefore be highly interesting to study more explicitly if individuals with HD have specific difficulties in experiencing social reward. This could also be highly relevant for clinical purposes, as it is well known that people with HD often present with anhedonia, affective flattening, depressive symptoms, and suicidal ideation. If difficulties in appreciating social interaction as rewarding were present in HD, such problems could be causally involved in negative affectivity, akin to findings in schizophrenia (Buck & Lysaker, 2013). On the other hand, patients with schizophrenia involved in the present study also presented with negative symptoms, suggesting that the reverse conclusion (i.e., anhedonia impacting one's willingness to engage in third‐party punishment) may not hold, because this clinical group performed in the DG much more similar to healthy controls.

The present study has several limitations. First, we cannot rule out that medication had some effect on economic decision‐making in HD patients (as well as in schizophrenia patients). Second, the duration of illness (onset of motor signs) was relatively short (also a strength, because cognitive decline was low), but it would be interesting to see if such aberrant behavior would already be present in pre‐manifest mutation carriers. Third, this is a purely behavioral study with no neurophysiological or neuroimaging correlates. Forth, the groups were not ideally matched for gender, nor was executive functioning examined across groups.

## 5 CONCLUSION

In summary, to our knowledge this is the first study to demonstrate alterations of HD patients' appreciation of rules of social exchange, particularly in relation to engagement in third‐party punishment. In our view, these interesting findings need to be taken further along the lines suggested above.

## CONFLICT OF INTEREST

The other authors declare that they have no conflict of interest.

## AUTHOR CONTRIBUTIONS

MB designed and conceptualized the research project; data acquisition was carried out by SMvH and CC; MB did the statistical analysis; MB, RH, and CS supervised the data acquisition; MB and SMvH wrote the first draft of the manuscript; all authors reviewed the manuscript.

### Peer Review

The peer review history for this article is available at https://publons.com/publon/10.1002/brb3.1908.

## Supporting information

Figure S1

Supplementary Material

## Data Availability

The data that support the findings of this study are available from the corresponding author upon reasonable request.
