# Supplementary figures and images for: Altered third‐party punishment in Huntington's disease: A study using neuroeconomic games
Source: Brain Behav. 2020 Oct 18;11(1):e01908. doi: 10.1002/brb3.1908 (PMC7821630; doi:10.1002/brb3.1908)

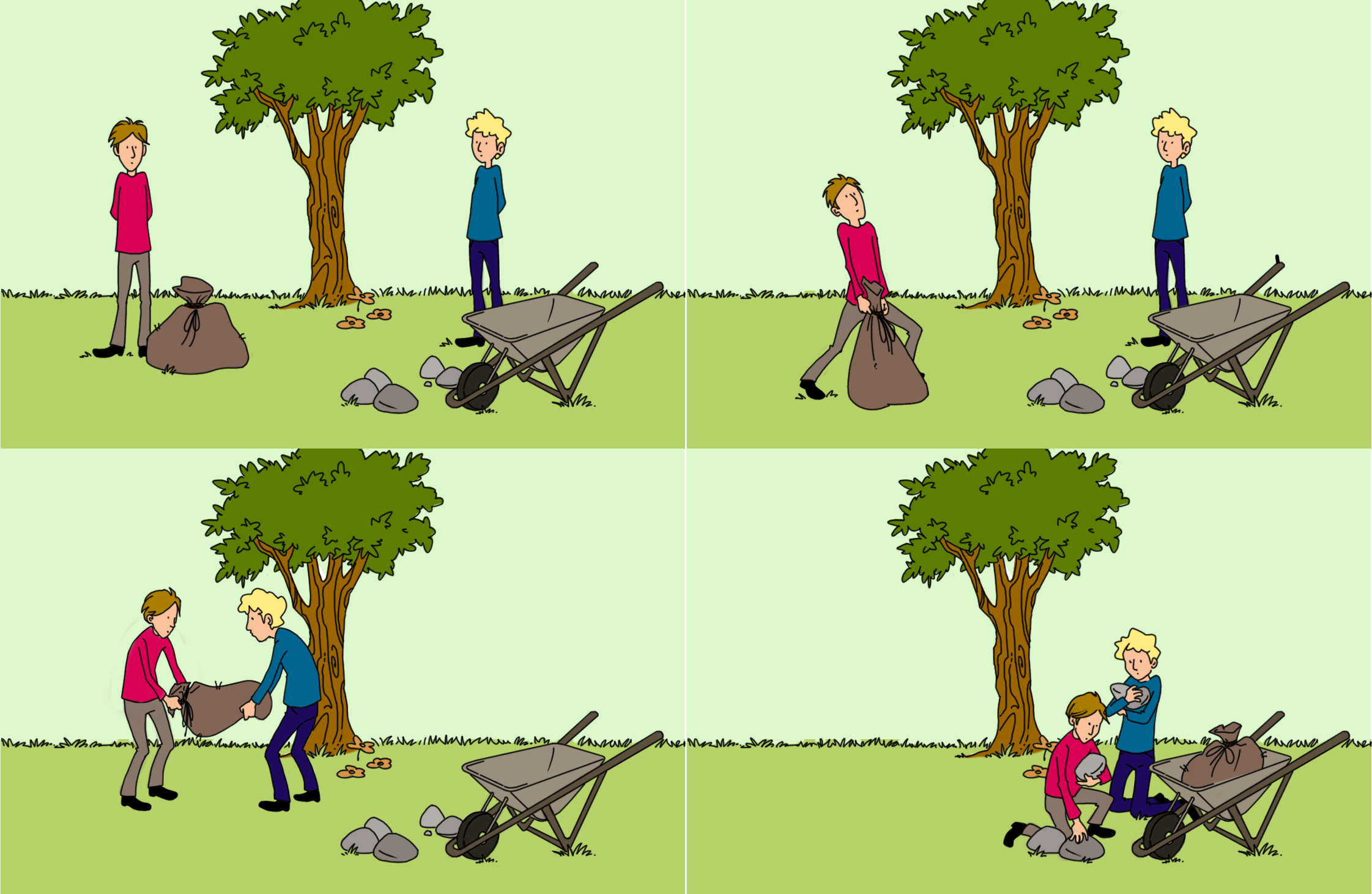

Supplement: Supplementary file 1 — Figure S1 [file BRB3-11-e01908-s001.tif]
