# Supplementary material for: Altered third‐party punishment in Huntington's disease: A study using neuroeconomic games
Source: Brain Behav. 2020 Oct 18;11(1):e01908. doi: 10.1002/brb3.1908 (PMC7821630; doi:10.1002/brb3.1908)
Supplement: Supplementary file 2 — Supplementary Material [file BRB3-11-e01908-s002.docx]

**Figure S1** Example of one of the six “theory of mind” cartoon stories.
